# PINCH1 Is Transcriptional Regulator in Podocytes That Interacts with WT1 and Represses Podocalyxin Expression

**DOI:** 10.1371/journal.pone.0017048

**Published:** 2011-02-24

**Authors:** Dan Wang, Yingjian Li, Chuanyue Wu, Youhua Liu

**Affiliations:** Department of Pathology, University of Pittsburgh School of Medicine, Pittsburgh, Pennsylvania, United States of America; University of Bergen, Norway

## Abstract

**Background:**

PINCH1, an adaptor protein containing five LIM domains, plays an important role in regulating the integrin-mediated cell adhesion, migration and epithelial-mesenchymal transition. PINCH1 is induced in the fibrotic kidney after injury, and it primarily localizes at the sites of focal adhesion. Whether it can translocate to the nucleus and directly participate in gene regulation is completely unknown.

**Methodology/Principal Findings:**

Using cultured glomerular podocytes as a model system, we show that PINCH1 expression was induced by TGF-β1, a fibrogenic cytokine that promotes podocyte dysfunction. Interestingly, increased PINCH1 not only localized at the sites of focal adhesions, but also underwent nuclear translocation after TGF-β1 stimulation. This nuclear translocation of PINCH1 was apparently dependent on the putative nuclear export/localization signals (NES/NLS) at its C-terminus, as deletion or site-directed mutations abolished its nuclear shuttling. Co-immunoprecipitation and pull-down experiments revealed that PINCH1 interacted with Wilms tumor 1 protein (WT1), a nuclear transcription factor that is essential for regulating podocyte-specific gene expression in adult kidney. Interaction of PINCH1 and WT1 was mediated by the LIM1 domain of PINCH1 and C-terminal zinc-finger domain of WT1, which led to the suppression of the WT1-mediated podocalyxin expression in podocytes. PINCH1 also repressed podocalyxin gene transcription in a promoter-luciferase reporter assay.

**Conclusion/Significance:**

These results indicate that PINCH1 can shuttle into the nucleus from cytoplasm in podocytes, wherein it interacts with WT1 and suppresses podocyte-specific gene expression. Our studies reveal a previously unrecognized, novel function of PINCH1, in which it acts as a transcriptional regulator through controlling specific gene expression.

## Introduction

Podocytes are highly differentiated glomerular visceral epithelial cells that play an essential role in the establishment of the glomerular filtration barrier, a structural apparatus that selectively restricts the filtration of different macromolecules in the blood stream on the basis of their sizes, shape and charge [1], [2]. The characteristic features of podocytes are their sophisticated foot processes, which are connected with the counterpart of the neighboring cells through specialized adhesion complexes known as slit diaphragms. Not surprisingly, podocyte dysfunction, as defined by foot process effacement/retraction and cell dedifferentiation, is one of the primary causes of proteinuria in a wide variety of human and experimental glomerular diseases, such as diabetic nephropathy, adriamycin nephropathy, and focal and segmental glomerulosclerosis (FSGS) [3]–[7].

The delicate morphology and function of podocytes are ultimately controlled by their unique transcriptional program in the nuclei. In that regard, WT1, the product of Wilms tumor gene 1, is a key nuclear transcription factor that plays a fundamental role in controlling the expression of major podocyte-specific genes such as podocalyxin in adult kidney [8]–[13]. WT1 is expressed early in embryonic kidney development and plays a crucial role in directing mammalian nephron formation, as homozygous mutations in WT1 result in embryonic lethality due to a failure in the development of kidneys [14], [15]. In adult kidney, WT1 expression is exclusively restricted to glomerular podocytes [16]. Based on these findings, WT1 is often utilized as a molecular marker for evaluating podocyte number and density under different circumstances [17]. However, how WT1 activity is regulated in podocytes is largely unknown.

PINCH1 (particularly interesting new cysteine-histidine rich protein 1) is an adaptor protein that plays an important role in regulating cell spreading, motility, epithelial-mesenchymal transition and matrix production [18]–[21]. Structurally, PINCH1 contains a tandem array of five LIN11, Isl1 and MEC-3 (LIM) domains that are involved in mediating protein-protein interactions, and a short C-terminal tail that harbors a putative leucine-rich nuclear export signal (NES) and overlapping basic nuclear localization signal (NLS) [21], [22]. As an adaptor protein, PINCH1 has been shown to interact with integrin-linked kinase (ILK) and promote the integrin signaling [23]–[26]. It also binds to Nck [27], another adaptor protein that links to nephrin [28], [29]. PINCH1 protein is predominantly localized at the focal adhesion sites of the periphery of spreading cells [20], a pattern overlapping with other focal adhesion proteins, such as ILK and paxillin. Earlier studies show that the affinity for binding of PINCH1 to ILK is reduced in cultured podocytes after injury induced by TGF-β1 [30]. However, it remains completely mysterious whether PINCH1 changes its sub-cellular localization after injury; and if so, what are the functional consequences in podocytes.

In this study, we demonstrate that PINCH1 is induced and undergoes nuclear translocation in podocytes after TGF-β1 treatment. Furthermore, we have shown that PINCH1 interacts with WT1, which leads to suppression of the WT1-mediated podocalyxin gene expression. Our data identify nuclear transcription factor WT1 as a novel binding partner for PINCH1, and provide novel insights into the mechanism of podocyte dysfunction under pathological conditions.

## Results

### Induction of PINCH1 expression by TGF-β1 in glomerular podocytes

**Figure 1:**
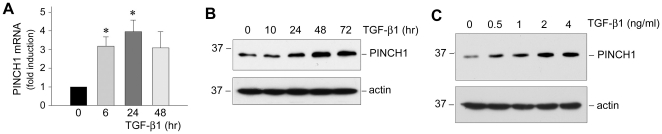
TGF-β1 induces PINCH1 mRNA and protein expression in human podocytes. *A*, Quantitative real-time RT-PCR reveals that TGF-β1 induced PINCH1 mRNA expression in a time-dependent manner. PINCH1 mRNA levels were assessed by quantitative real-time RT-PCR in human podocytes after TGF-β1 treatment (2 ng/ml) for various periods of time as indicated. Relative PINCH1 mRNA levels (fold induction over the controls) were reported after normalization with β-actin, and presented as mean ± SEM of three experiments. *P<0.05 versus controls. *B* and *C*, Western blot analyses demonstrate that TGF-β1 induced PINCH1 protein expression in a time- and dosage-dependent manner. Human podocytes were treated with a fixed amount of TGF-β1 (2 ng/ml) for various periods of time as indicated (B) or with various concentrations of TGF-β1 for 48 h (C). Total cell lysates were immunoblotted with specific antibodies against PINCH1 and actin, respectively.

We first examined the expression of PINCH1 in human podocytes after incubation with TGF-β1, a potent fibrogenic cytokine that is shown to induce podocyte dysfunction in a wide variety of proteinuric chronic kidney diseases [3], [31]. As shown in Figure 1A, PINCH1 mRNA was significantly induced in cultured human podocytes following TGF-β1 stimulation, as demonstrated by a quantitative real-time RT-PCR assay. This induction of PINCH1 mRNA started at 6 h, reached the peak at 24 h and sustained at least to 48 h after TGF-β1 treatment. Consistent with the mRNA expression, PINCH1 protein expression was also induced by TGF-β1 in podocytes, as illustrated by Western blot analyses of the whole cell lysates (Figure 1, B and C). TGF-β1 induced PINCH1 protein in a time-dependent manner, which started at 24 h and sustained at least to 72 h after treatment, time points that significantly lagged behind the mRNA induction. The induction of PINCH1 expression also occurred in a dose-dependent fashion; and TGF-β1 induced its protein level at the concentration as low as 0.5 ng/ml, which reached the peak at 2 ng/ml.

### Nuclear translocation of PINCH1 in podocytes after TGF-β1 stimulation

**Figure 2:**
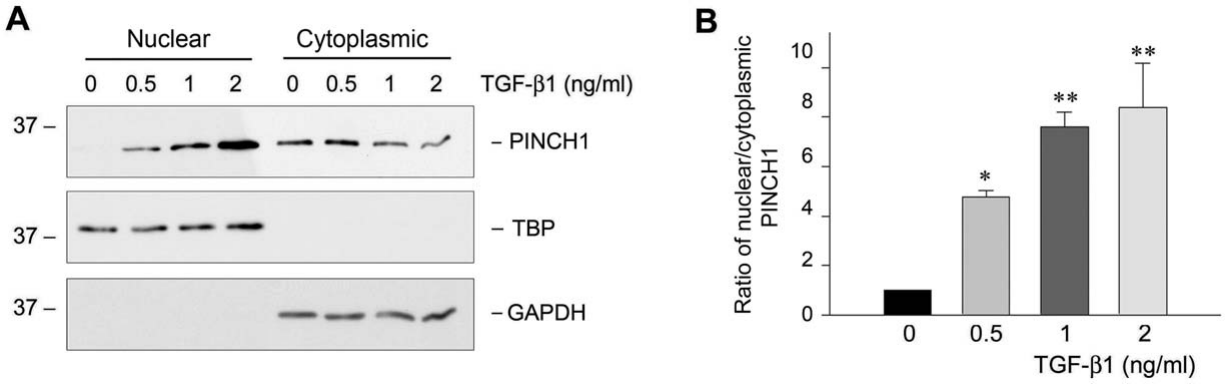
TGF-β1 induces nuclear translocation of PINCH1 in human podocytes. *A*, Western blot analyses show marked induction of PINCH1 protein in the nuclei of podocytes after TGF-β1 treatment. Podocytes were incubated with TGF-β1 at various concentrations as indicated for 72 h. Nuclear and cytoplasmic preparations were made and immunoblotted with specific antibodies against PINCH1, TBP and GAPDH, respectively. *B*, Graphic presentation shows the relative ratio of the nuclear/cytoplasmic PINCH1 protein in podocytes after TGF-β1 treatment. The value of the nuclear/cytoplasmic ratio of PINCH1 in the control group was set as 1.0. Data are presented as mean ± SEM of four experiments. *P<0.05, **P<0.01 versus controls.

As an adaptor protein that binds to ILK, PINCH1 is primarily localized at the cell focal adhesion sites [20]. However, we found that PINCH1 was increasingly accumulated in the nuclei of podocytes after TGF-β1 stimulation. Subcellular fractionation experiments revealed that nuclear PINCH1 was dramatically increased in a dose-dependent fashion, while its levels in the cytoplasmic preparation slightly declined after TGF-β1 treatment (Figure 2A). Quantitative determination of the ratio of nuclear/cytoplasmic PINCH1 in podocytes after TGF-β1 treatment is presented in Figure 2B. These results indicate that TGF-β1 not only induces PINCH1 expression, but also triggers it to undergo nuclear translocation in podocytes.

**Figure 3:**
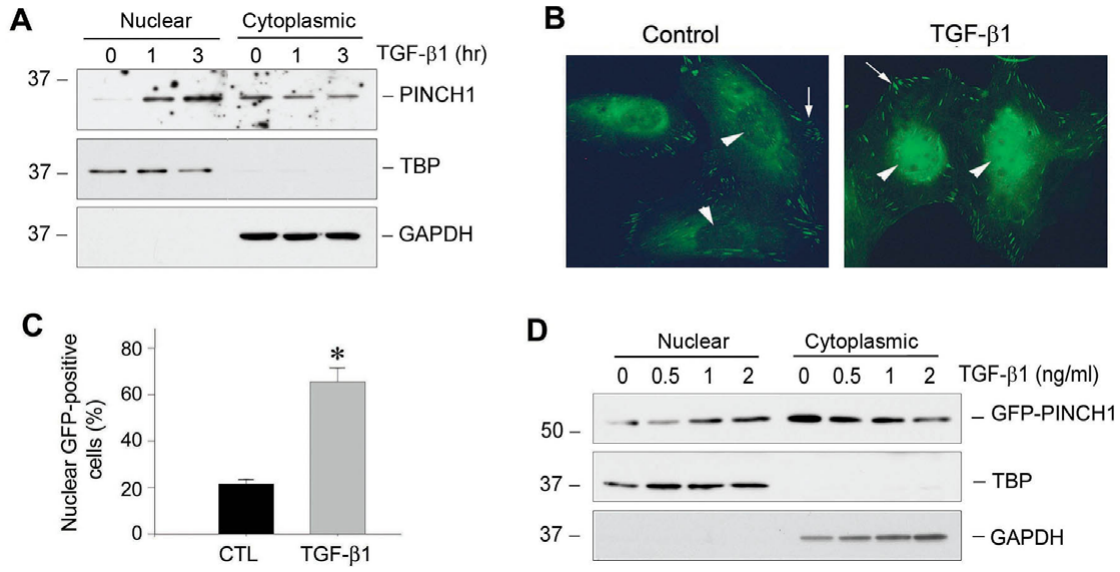
PINCH1 nuclear translocation induced by TGF-β1 is regulated by a mechanism independent of its abundance. *A*, Western blot analyses show nuclear accumulation of endogenous PINCH1 protein in podocytes after a short period of TGF-β1 treatment. Podocytes were incubated with TGF-β1 (2 ng/ml) for short periods of time as indicated. Nuclear and cytoplasmic proteins were separated and immunoblotted with specific antibodies against PINCH1, TBP and GAPDH, respectively. *B*, Representative micrographs show the sub-cellular localization of GFP-PINCH1 fusion protein in control or TGF-β1-treated podocytes. Podocytes were transfected with GFP-PINCH1 expression plasmid driven under CMV promoter for 24 h, and then incubated with TGF-β1 for additional 24 h at various concentrations as indicated. Arrows indicate the localization of PINCH1 in the focal adhesion, while arrowheads denote the nuclear localization of PINCH1. *C*, Graphic presentation shows the percentage of the podocytes with positive nuclear GFP protein after TGF-β1 treatment. Data are presented as mean ± SEM of three experiments. *P<0.05 versus controls. *D*, Western blot analyses show an induction of GFP-PINCH1 protein in the nuclei of podocytes by TGF-β1. Nuclear and cytoplasmic proteins were separated and immunoblotted with specific antibodies against PINCH1, TBP and GAPDH, respectively.

To rule out the possibility that an increased nuclear accumulation of PINCH1 is a consequence of its over-expression, we next tested whether TGF-β1 directly promotes the nuclear translocation of endogenous PINCH1. To this end, we examined the nuclear accumulation of PINCH1 after a short incubation with TGF-β1, as it did not significantly induce PINCH1 protein expression until 24 h of incubation (Figure 1B). As shown in Figure 3A, incubation with TGF-β1 for 1 to 3 h was sufficient to induce the nuclear translocation of endogenous PINCH1, indicating that TGF-β1-induced nuclear translocation of PINCH1 is regulated by a mechanism independent of its abundance.

To further confirm the nuclear translocation of PINCH1, we constructed an expression vector of GFP-PINCH1 fusion protein driven under CMV promoter. After transfection of this GFP-PINCH1 expression vector for 24 h, podocytes were treated with TGF-β1 for 24 h. We examined GFP-tagged PINCH1 subcellular distribution in podocytes using microscopy. As shown in Figure 3B, under basal conditions, GFP-PINCH1 was mainly localized in the peri-nuclear region of the cytoplasm, as well as at the focal adhesion sites in the periphery of spreading podocytes (Figure 3B, left panel, arrows), but little in the nuclei (Figure 3B, left panel, arrowhead). However, GFP-tagged PINCH1 diminished in the peri-nuclear region and was clearly accumulated in the nuclei of podocytes after TGF-β1 treatment (Figure 3B, right panel, arrowhead), although it remained present at the focal adhesion sites (Figure 3B, right panel, arrow). Quantitative calculation showed more than 60% of podocytes that exhibited GFP-PINCH1 in the nuclei after TGF-β1 stimulation, a significant increase over the controls (Figure 3C). To analyze this in a more quantitative way, we assessed nuclear and cytoplasmic GFP-PINCH1 abundance by Western blotting. As shown in Figure 3D, nuclear GFP-PINCH1 was increased, while its level in the cytoplasm declined, thereby leading to a significant shift in the ratio of nuclear/cytoplasmic GFP-PINCH1 after TGF-β1 treatment. Since GFP-tagged PINCH1 is controlled under CMV promoter, which is not regulated by TGF-β1, these results indicate that TGF-β1 is able to induce nuclear translocation of PINCH1 apparently by an active process independent of its protein abundance.

### Nuclear translocation of PINCH1 requires its putative NES/NLS motif

**Figure 4:**
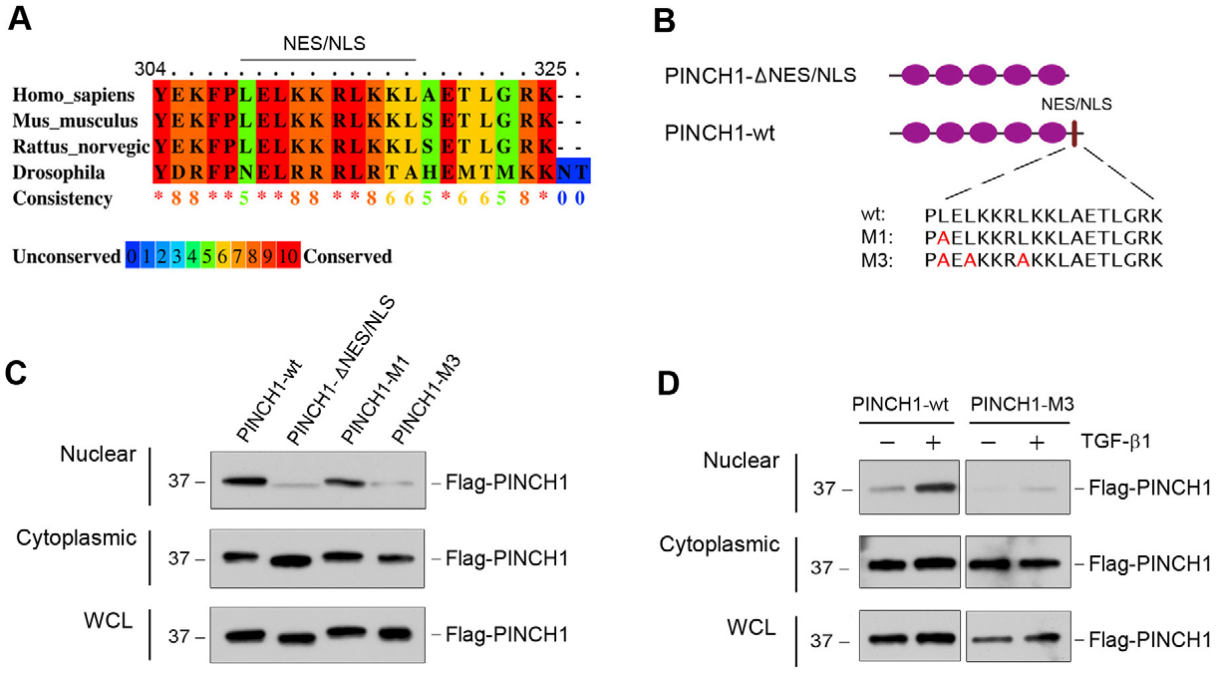
Subcellular localization of PINCH1 is dictated by a putative motif in its C-terminus. *A*, Amino acid sequence comparison reveals a conserved, overlapped, putative NES/NLS motif in the C-terminus of PINCH1 among different species including human, mouse, rat, and *Drosophila*. Alignments of the deduced amino acid sequences were performed by using PRALINE program. Color bar is a strength histogram that denotes the least to most conserved amino acids (least: dark blue, light blue, green, orange, red: most). *B*, Schematic diagram shows the structural domains of PINCH1 and construction of various PINCH1 mutants. Purple ovals indicate the five LIM domains. The position of a putative NES/NLS motif is shown by a bar, and the sequences of wild-type and mutant NES/NLS are given. *C*, Deletion or mutation of the putative NES/NLS blocks nuclear translocation of PINCH1 in podocytes. Human podocytes were transfected for 48 h with Flag-tagged wild-type PINCH1 (pFlag-PINCH1-wt), truncated PINCH1 without NES/NLS (p-Flag-PINCH1-ΔNES/NLS), PINCH1 with single amino acid mutation in the NES/NLS motif (pFlag-PINCH1-M1) and PINCH1 with three amino acids mutation in the NES motif (pFlag-PINCH1-M3), respectively. Nuclear and cytoplasmic proteins were separated and immunoblotted with antibodies against Flag. *D*, TGF-β1 treatment fails to induce nuclear translocation of PINCH1 with mutant NES/NLS. Podocytes were transfected with wild-type PINCH1 (pFlag-PINCH1-wt) or mutant PINCH1 (pFlag-PINCH1-M3) for 48 h, respectively, and then treated TGF-β1 (2 ng/ml) for 3 h. Nuclear and cytoplasmic proteins were separated and immunoblotted with antibodies against Flag.

Bioinformatics analysis revealed that PINCH1 harbors a leucine-rich, putative nuclear export signal (NES) and an overlapping nuclear localization signal (NLS), consisting of charged, mostly basic amino acids such as lysine and arginine in its C-terminus, as previously reported [22]. Alignment of PINCH1 sequences derived from different species indicated that this region was highly conserved during evolution (Figure 4A). To test whether this putative NES/NLS is responsible for mediating cytoplasmic/nuclear shuttling of PINCH1, we generated the truncated, Flag-tagged PINCH1 expression vector (pFlag-PINCH1-ΔNES/NLS) in which the putative NES/NLS was deleted. In addition, we also constructed a Flag-tagged, wild-type PINCH1 (pFlag-PINCH1-wt), as well as two mutant PINCH1 expression vectors (pFlag-PINCH1-M1 and pFlag-PINCH1-M3) by site-directed mutagenesis, as illustrated in Figure 4B. These mutant PINCH1 vectors contained either one or three leucine to alanine (L/A) point mutations in the NES/NLS motif, respectively (Figure 4B). As shown in Figure 4C, deletion of putative NES/NLS almost completely blocked nuclear translocation of PINCH1, compared with the wild type PINCH1 controls. Furthermore, mutations at three leucine positions (M3) in the NES/NLS motif also rendered PINCH1 unable to undergo nuclear shuttling (Figure 4C). However, a single mutation at one leucine position (M1) only marginally, if any, reduced the nuclear accumulation of PINCH1 (Figure 4C). We further examined whether the putative NES/NLS is required for TGF-β1-induced nuclear translocation of PINCH1 by transfecting either wild-type or mutant PINCH1. As demonstrated in Figure 4D, TGF-β1 could induce wild-type, but not mutant (M3), PINCH1 nuclear translocation in podocytes. Therefore, it appears that the putative NES/NLS motif is required for mediating nuclear shuttling of PINCH1 under both basal and TGF-β1-stimulated conditions.

### PINCH1 interacts with nuclear transcription factor WT1

**Figure 5:**
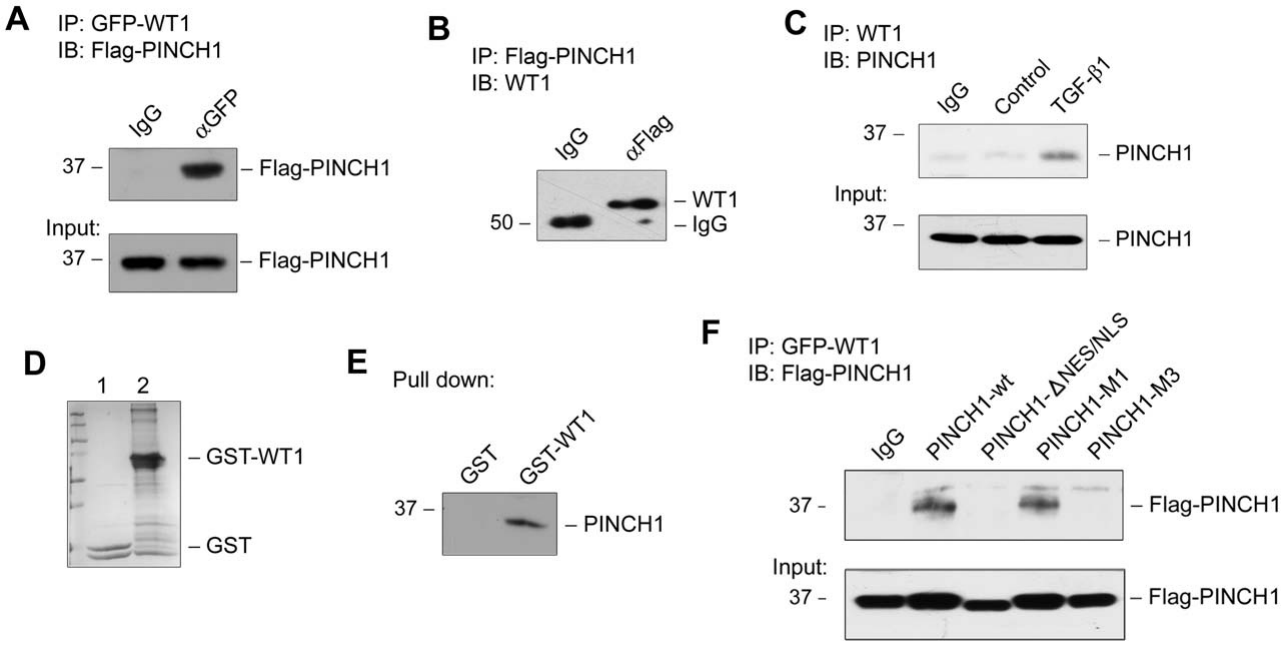
PINCH1 physically interacts with nuclear transcription factor WT1 in human podocytes. *A*, Co-immunoprecipitation demonstrates a complex formation between PINCH1 and WT1. Human podocytes were transfected with Flag-PINCH1 and GFP-WT1 for 48 h. Cell lysates were immunoprecipitated with specific antibody against GFP, followed by immunoblotting with antibody against Flag. IgG, control rabbit IgG. *B*, Co-immunoprecipitation shows a complex formation between PINCH1 and WT1. Human podocytes were transfected with Flag-PINCH1 for 48 h. Cell lysates were immunoprecipitated with specific antibody against Flag, followed by immunoblotting with antibody against endogenous WT1. IgG, control mouse IgG. *C*, Endogenous WT1 and PINCH1 interaction after TGF-β1 treatment in podocytes. Human podocytes were treated with TGF-β1 (2 ng/ml) for 24 h. Cell lysates were immunoprecipitated with anti-WT1 antibody, followed by immunoblotting with anti-PINCH1. *D*, Coomassie blue staining shows the purified GST tagged proteins expressed in bacterial BL21. Lane 1, purified GST. Lane 2, purified GST-WT1 fusion protein. *E*, Pull-down assay revealed a complex formation between PINCH1 and WT1. Purified GST-WT1 protein as well as control GST protein was incubated with podocyte lysate overnight, followed by immunoblotting with antibody against PINCH1. *F*, Deletion or mutation of the putative NES/NLS motif prevents PINCH1/WT1 interaction in podocytes. Human podocytes were transfected for 48 h with GFP-tagged WT1 (pGFP-WT1) and Flag-tagged wild-type PINCH1 (pFlag-PINCH1-wt), truncated PINCH1 without NES/NLS (p-Flag-PINCH1-ΔNES/NLS), PINCH1 with single amino acid mutation in the NES/NLS motif (pFlag-PINCH1-M1) and PINCH1 with three amino acids mutation in the NES motif (pFlag-PINCH1-M3), respectively. Cell lysates were immunoprecipitated with anti-GFP antibody, followed by immunoblotting with anti-Flag antibody. An aliquot of cell lysates were immunoblotted with anti-Flag antibody as input.

The finding that PINCH1 can shuttle into the nucleus prompted us to investigate its potential function in podocytes. In view of the structural characteristics of PINCH1, which contains five LIM domains that mediate protein-protein interactions, we reasoned that PINCH1 might interact with other nuclear proteins that are important for podocyte biology. Along this line, we found that PINCH1 could interact with WT1, a transcription factor that is exclusively expressed in podocytes in adult kidney. As shown in Figure 5A, when Flag-tagged PINCH1 and GFP-tagged WT1 were co-expressed in podocytes, PINCH1 could be detected in the immunocomplexes precipitated by anti-GFP antibody. In reciprocal experiments, after transfection of podocytes with Flag-tagged PINCH1 expression vector, endogenous WT1 was found in the immunocomplexes precipitated with anti-Flag antibody (Figure 5B). These interactions between PINCH1 and WT1 appeared specific, as replacing specific antibodies with control IgG did not result in any binding (Figure 5, A and B). Furthermore, physical interaction between endogenous PINCH1 and WT1 was detectable in podocytes after TGF-β1 stimulation (Figure 5C), suggesting that PINCH1/WT1 complex formation actually occurs in pathophysiologically relevant conditions.

To further confirm the specificity of PINCH1/WT1 interaction, we employed a GST-fusion protein pull down experiment to examine the interaction between PINCH1 and WT1. To this end, we generated a GST-WT1 fusion protein using a bacterial expression system. As shown in Figure 5D, GST-WT1 fusion protein as well as GST control protein was purified. When these purified proteins were immobilized on glutathione-agarose beads and incubated with podocyte lysates, PINCH1 was pulled down and detected in the assay (Figure 5E), indicating a specific interaction between PINCH1 and WT1.

We also tested whether deletion or mutation of the putative NES/NLS motif prevents PINCH1/WT1 interaction in podocytes. As shown in Figure 5F, when podocytes were transfected with GFP-tagged WT1 and Flag-tagged wild-type PINCH1, PINCH1/WT1 interaction was readily detectable by co-immunoprecipitation. However, no or little PINCH1/WT1 interaction was observed when PINCH1 mutants with either deletion of the NES/NLS (PINCH1-ΔNES/NLS) or three amino acid mutations within this motif (PINCH1-M3) were co-transfected with GFP-tagged WT1. These data suggest that nuclear translocation of PINCH1 is a prerequisite for its interaction with WT1 in podocytes.

### Delineation of the structural domains mediating PINCH1/WT1 interaction

**Figure 6:**
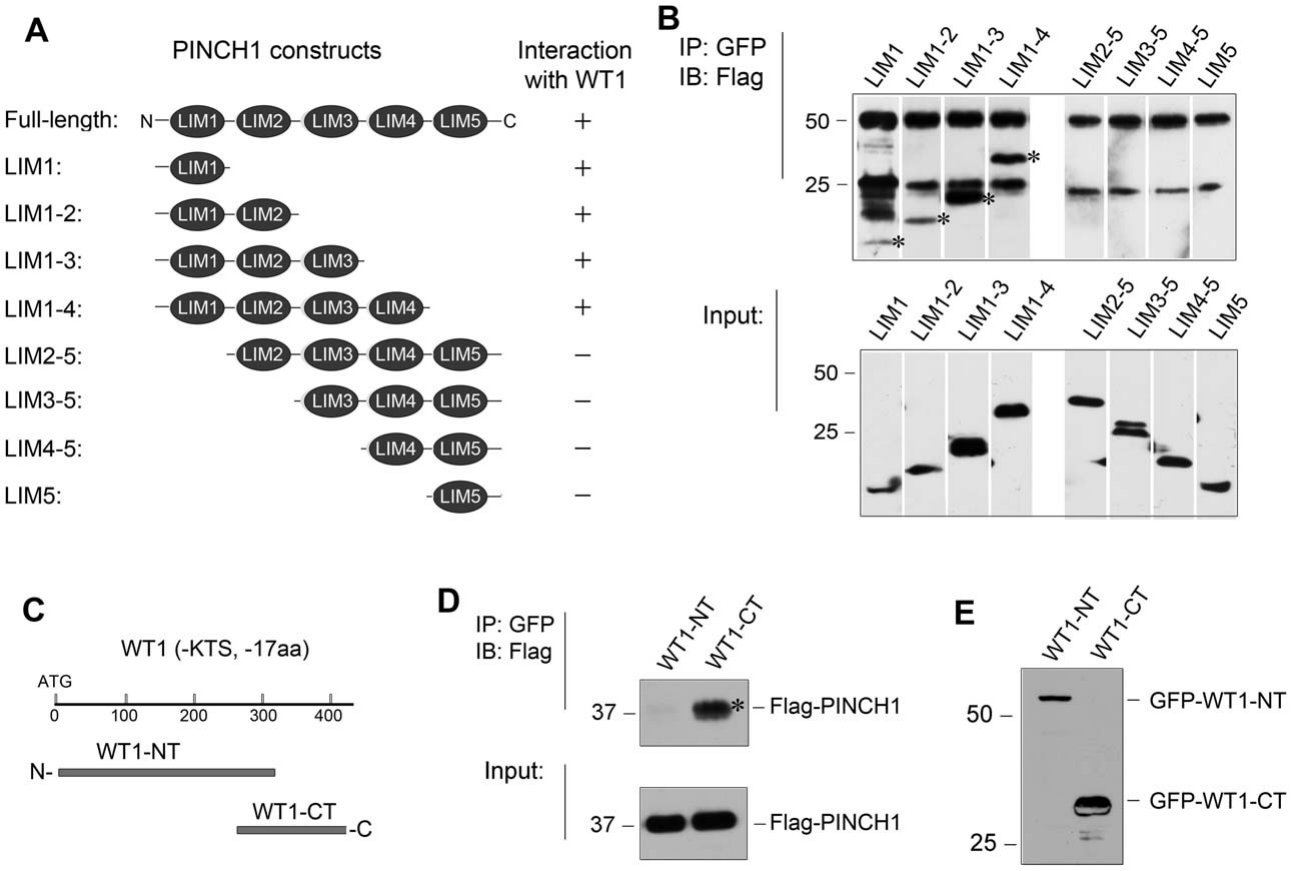
Delineation of the structural domains that mediate PINCH1/WT1 interaction. *A*, Schematic diagram shows the construction of various Flag-tagged PINCH1 expression vectors containing different LIM domains. *B*, Co-immunoprecipitation indicates that the LIM1 domain of PINCH1 mediates the interaction with WT1. Human podocytes were co-transfected with various Flag-tagged PINCH1 LIM domains and GFP-WT1 for 48 h. Cell lysates were immunoprecipitated with specific antibody against GFP, followed by immunoblotting with antibody against Flag. Asterisk (*) indicate positive interactions. *C*, Diagram shows the construction of GFP tagged WT1 fragments. WT1 used is the isoform without KTS and 17 aa encoded by exon 5 (WT1, -KTS, -17 aa). WT1-NT, N-terminal fragment of WT1 (1–315 aa); WT1-CT, C-terminal fragment of WT1 (280–429 aa). *D*, Co-immunoprecipitation shows that PINCH1 interacted with WT1-CT, but not WT1-CT. Human podocytes were transfected with Flag-PINCH1 and either GFP-tagged WT1-NT or GFP-tagged WT1-CT for 48 h. Cell lysates were immunoprecipitated with specific antibody against GFP, followed by immunoblotting with anti- Flag. *E*, Expression of GFP-tagged WT1 fragments in podocytes after transfection were confirmed by Western blot analysis.

To define the structural domains responsible for mediating PINCH1/WT1 interaction, we generated a series of Flag-tagged, truncated PINCH1 expression vectors containing different LIM domains (Figure 6A). After these constructs were co-transfected with GFP-tagged WT1 expression vector into podocytes, physical interaction between different domains of PINCH1 and WT1 were assessed by co-immunoprecipitation. As shown in Figure 6B, all truncated PINCH1 proteins that contained LIM1 domain were detected in the immunocomplexes precipitated by anti-GFP antibody, whereas those PINCH1 proteins without LIM1 were not found in the immunoprecipitates under the same conditions. These results indicate that the LIM1 domain of PINCH1 mediates its interaction with WT1 (Figure 6B).

We also sought to determine the structural domain of WT1 that is involved in its interaction with PINCH1. To this end, we generated two GFP-tagged expression vectors that contained either N-terminal, proline/glutamine-rich regulatory domain (1-315 aa) (WT1-NT) or C-terminal, DNA-binding domain (280-429 aa) (WT1-CT) containing four zinc fingers of the Kruppel-type (Figure 6C), respectively. When these expression vectors were co-transfected with Flag-tagged PINCH1 construct in podocytes, potential interaction between truncated WT1 and PINCH1 was examined by co-immunoprecipitation. As shown in Figure 6, D and E, PINCH1 was only detected in the immunocomplexes in podocytes over-expressing GFP-tagged C-terminus of WT1 (WT1-CT), but not in the cells transfected with expression vector encoding N-terminus of WT1 (WT1-NT). Therefore, it appears clear that the C-terminal zinc-finger domains of WT1 mediate its interaction with PINCH1.

### PINCH1 represses WT1-mediated podocalyxin expression

**Figure 7:**
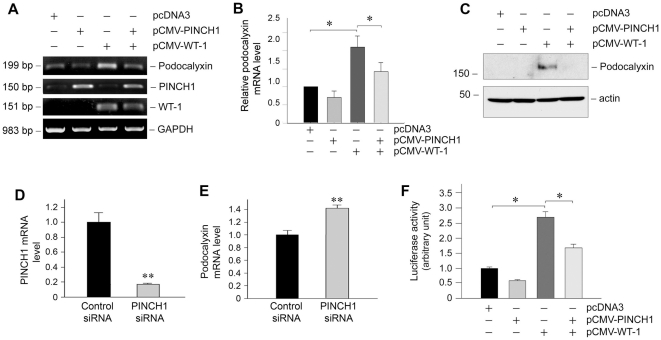
PINCH1 blocks WT1-mediated podocalyxin expression in human podocytes. *A*, RT-PCR analyses demonstrate that PINCH1 blocked WT1-stimulated podocalyxin mRNA expression in podocytes. Cells were transfected with expression vectors for PINCH1, WT1 or both, respectively. RT-PCR amplification of housekeeping GAPDH was performed in an identical manner to serve as controls. *B*, Graphic presentation shows the relative PINCH1 mRNA abundance in different groups after normalization with GAPDH. Data are presented as mean ± SEM of three independent experiments. *P<0.05. *C*, Western blot analyses show that PINCH1 blocked WT1-mediated podocalyxin protein expression. Human podocytes were transfected with different plasmids as indicated for 48 h. Total cell lysates were immunoblotted with specific antibodies against podocalyxin and actin, respectively. *D* and *E*, Knockdown of PINCH1 in podocytes promotes podocalyxin expression. Human podocytes were transfected with either control or PINCH1-specific siRNA. The expression of PINCH1 (*D*) and podocalyxin (*E*) was assessed by quantitative RT-PCR. **P<0.01 (n = 3). *F*, PINCH1 represses WT1-activated podocalyxin gene promoter activity. Human podocytes were co-transfected with different plasmids as indicated with luciferase- podocalyxin gene promoter reporter construct (pGL3-podocalyxin) for 48 h. Equal amounts of DNA were present in each transfection. Data are presented as mean ± SEM of three independent experiments. *P<0.05.

To examine the potential consequence of PINCH1/WT1 interaction, we investigated the effects of PINCH1 on WT1-mediated gene expression. Podocalyxin, a transmembrane protein that plays a crucial role in the maintenance of podocyte morphology and foot processes [32], is well characterized as a WT1 target gene. Indeed, we found that ectopic expression of WT1 in podocytes significantly induced podocalyxin mRNA and protein expression (Figure 7, A through C). However, co-transfection of PINCH1 largely abolished WT1-mediated podocalyxin induction. Of note, PINCH1 had the tendency, although not significantly, to inhibit basal podocalyxin mRNA expression in the absence of exogenous WT1 (Figure 7, A and B), suggesting that podocalyxin is also controlled by endogenous WT1 in podocytes. Likewise, knockdown of PINCH1 increased podocalyxin mRNA expression in podocytes (Figure 7, D and E). Therefore, it becomes clear that PINCH1 interacts with WT1, which leads to suppression of the WT1-mediated gene expression in podocytes.

We further investigated the effects of PINCH1 on podocalyxin gene transcription using a promoter-luciferase reporter assay. A podocalyxin promoter luciferase reporter vector containing a putative WT1 response element at the nucleotide positions −1213 to −1227 was constructed, as previously reported [10]. We found that co-transfection with WT1 expression vector significantly stimulated the podocylyxin promoter luciferase activity in podocytes, compared to empty vector pcDNA3 (Figure 7F). However, when PINCH1 was co-expressed, the WT1-mediated podocalyxin promoter-luciferase activity was suppressed, compared with transfection with WT1 alone (Figure 7F). Therefore, consistent with the suppression of podocalyxin protein and mRNA, PINCH1 also abolishes the WT1-mediated the transcription of podocalyxin gene.

## Discussion

PINCH1 is an adaptor/scaffolding protein that normally localizes at focal adhesion sites. In this study, we provide evidence demonstrating that PINCH1 also functions as a transcriptional regulator by interacting with nuclear WT1, a podocyte-specific transcription factor that plays a pivotal role in the establishment and maintenance of the unique differentiated features of podocytes in adult kidney. We show that PINCH1 is up-regulated in podocytes after stimulation with TGF-β1 and translocates into the nucleus, wherein it binds to WT1 and suppresses the WT1-mediated gene transcription. Our results uncover a novel function of PINCH1, in which it acts as a transcriptional regulator through controlling specific gene expression in podocytes.

Given the ability of PINCH1 to undergo nuclear translocation, it is conceivable that there are possible three subcellular (nuclear, cytoplasmic and focal adhesion-associated) pools of PINCH1 in podocytes. Each specific pool of PINCH1 may have different functions. While the focal adhesion-associated PINCH1 may play a critical role in modulating cell adhesion, cell shape and survival [33], we show here that nuclear PINCH1 is instrumental in regulating gene transcription via its interaction with WT1. Cellular stress/injury after TGF-β1 treatment accelerates the rate of its nuclear shuttling, resulting in an increased accumulation of PINCH1 in the nuclei, but it does not appear to significantly affect the focal adhesion-associated PINCH1 (Figure 3B). Nuclear accumulation of PINCH1 is unlikely a passive consequence of an increased overall level of its protein, since TGF-β1 also promotes nuclear translocation of PINCH1 after a short incubation (1–3 h) (Figure 3A) when significant PINCH1 induction was not evident (Figure 1B). Not surprisingly, the putative NES/NLS motif in its C-terminus of PINCH1 is functionally important and obligatory for mediating its nuclear translocation, as deletion or site-directed mutations of this motif effectively prevents its nuclear shuttling (Figure 4) and its interaction with WT1 (Figure 5) in podocytes. Such a PINCH1 shuttling between cytoplasm and nucleus is also reported in Schwann cells after chronic constriction injury in adult rats [22]. Consistently, several other LIM-containing proteins are found to be able to undergo nuclear shuttling [34]–[36]. In this context, it is reasonable to conclude that PINCH1 is able to translocate into the nucleus in response to injury, thereby initiating new protein-protein interactions and participating in the control of gene transcription in diverse circumstances.

One of the novel findings in the present study is the identification of WT1 transcription factor as the binding partner for PINCH1 in the nuclei. Through defining the molecular details of PINCH1/WT1 interaction, we show that the LIM1 domain of PINCH1 mediates its interaction with WT1, whereas the C-terminal zinc-finger domains of WT1 are responsible for its binding to PINCH1 (Figure 6). Because WT1, a key transcription factor that is exclusively expressed in glomerular podocytes in adult kidney, plays a critical role in establishing the unique features of podocytes by inducing specific gene expression, such a PINCH1/WT1 interaction likely has a detrimental consequence. Indeed, endogenous PINCH1/WT1 interaction actually occurs in podocytes after TGF-β1 stimulation (Figure 5C). Similarly, interaction between WT1 and the WT1-interacting protein (WTIP), another LIM-containing protein, is previously shown to lead to the suppression of WT1-mediated gene expression and podocyte dysfunction [34], [35], [37].

PINCH1 nuclear shuttling and subsequent interaction with WT1 could presumably influence the WT1-mediated gene expression in podocytes. In that regard, it is interesting to reveal that PINCH1 regulates the expression of podocalyxin, a well-characterized podocyte-specific protein that is transcriptionally controlled by WT1. Earlier *in vivo* and *in vitro* studies demonstrate that WT1 level and activity directly dictate podocalyxin expression in glomerular podocytes [10], [13], [32]. Indeed, ectopic expression of WT1 in cultured podocytes induces podocalyxin mRNA and protein expression (Figure 7). Given that PINCH1 binds to the zinc-finger domains of WT1, it is not unexpected that over-expression of PINCH1 abolishes WT1-mediated podocalyxin expression, while knockdown of PINCH1 induced podocalyxin expression (Figure 7). Podocalyxin is a CD34-related, transmembrane, sialoglycoprotein that contains a highly charged cytoplasmic tail [32]. It is connected to the cortical actin cytoskeleton via ezrin and Na^+^/H^+^-exchanger regulatory factor 2 (NHERF2) and plays an essential role in maintaining the foot process structure and filtration function. Disruption of podocalyxin/NHERF2/ezrin/actin interactions leads to pathologic conditions associated with changes in podocyte foot processes [38]. Consistently, podocalyxin-deficient mice fail to form foot processes and slit diaphragms and die within 24 h after birth with anuric renal failure [39]. Therefore, suppression of WT1-medated gene expression by PINCH1 could be a potential pathway leading to podocyte dysfunction.

In summary, we have shown that PINCH1 undergoes nuclear shuttling in podocytes after TGF-β1 stimulation. Nuclear PINCH1 via its LIM1 domain interacts with a new partner WT1. By interacting with the zinc finger domains of WT1, PINCH1 effectively blocks WT1-mediated gene transcription. These studies provide a proof of principal that PINCH1 can function as a transcriptional regulator by regulating specific gene expression.

## Materials and Methods

### Cell culture and treatment

The conditionally immortalized human podocyte cell line was kindly provided by Dr. M. Saleem (University of Bristol, Bristol, UK), as described previously [16], [40]. To propagate podocytes, cells were cultured at 33°C in RPMI-1640 medium supplemented with 10% fetal bovine serum and a mixture of insulin, transferrin and sodium selenite (ITS) (I3146; Sigma, St. Louis, MO). To induce differentiation, podocytes were grown under nonpermissive conditions at 37°C to inactivate the SV40 large T antigen. Podocytes were treated with recombinant TGF-β1 at the concentration of 2 ng/ml, unless otherwise indicated. For some studies, podocytes were transiently transfected with various PINCH1 expression vectors by using Lipofectamine 2000 reagent (Invitrogen, Carlsbad, CA), as described previously [41].

### Construction of various expression vectors

Various expression vectors with different epitope-tags were constructed using routine molecular cloning techniques. Flag- and GFP-tagged PINCH1 expression vectors (pFlag-PINCH1 and pGFP-PINCH1), as well as GFP-tagged WT1 (pGFP-WT1) were constructed in the pcDNA3-based expression vector under the control of CMV promoter. The expression vector for WT1 (-KTS) without KTS was kindly provided by Dr. D. Haber (Massachusetts General Hospital, Charlestown, MA). The expression vector for truncated PINCH1 without NES/NLS (pFlag-PINCH1-ΔNES/NLS) was constructed by PCR using the wild-type PINCH1 expression plasmid (pFlag-PINCH1-wt) as a template. The expression vectors for mutant PINCH1 with either a single amino acid mutation (pFlag-PINCH1-M1) or three amino acid mutations in the NES/NLS motif (pFlag-PINCH1-M3) were made by using QuikChange II XL site-directed mutagenesis kit (Stratagene, La Jolla, CA). The correct sequences of different expression vectors were confirmed by sequencing at the DNA Sequencing Core Facility of the University of Pittsburgh.

### RT-PCR and real-time PCR

Total RNA was extracted using the TRIzol RNA isolation system (Invitrogen, Carlsbad, CA). The first strand of cDNA was synthesized using 2 µg of RNA in 20 µl of reaction buffer by reverse transcription using AMV-RT (Promega, Madison, WI) and random primers at 42°C for 30 min. PCR was carried out using a standard PCR protocol with 1 µl aliquot of cDNA, HotStarTaq polymerase (Qiagen, Valencia, CA) and specific primer pairs. The sequences of the primer pairs were as follows: PINCH1, 5′ CCG CTG AGA AGA TCG TGA AC 3′ (sense) and 5′ GGG CAA AGA GCA TCT GAA AG 3′ (anti-sense); podocalyxin, 5′ GAG CAG TCA AAG CCA CCT TC 3′ (sense) and 5′ TGG TCC CCT AGC TTC ATG TC 3′ (anti-sense); WT1, 5′ GCG GAG CCC AAT ACA GAA TA 3′ (sense) and 5′ TTA TTG CAG CCT GGG TAA GC 3′ (anti-sense); GAPDH, 5′ TGA AGG TCG GAG TCA ACG GAT TTG GT 3′ (sense) and 5′ CAT GTG GGC CAT GAG GTC CAC CAC 3′ (anti-sense); β-actin, 5′ AGG CAT CCT CAC CCT GAA GTA 3′ (sense) and 5′ CAC ACG CAG CTC ATT GTA GA 3′ (anti-sense). For quantitative determination of mRNA levels, a real-time RT-PCR was performed on ABI PRISM 7000 Sequence Detection System (Applied Biosystems, Foster City, CA), as described previously [42]. The PCR reaction mixture in a 25 µl volume contained 12.5 µl of 2x SYBR Green PCR Master Mix (Applied Biosystems), 10 µl of diluted RT product (1∶10), and 0.5 µM sense and antisense primer sets. PCR reaction was run by using standard conditions [42]. After sequential incubations at 50°C for 2 min and 95°C for 10 min, respectively, the amplification protocol consisted of 40 cycles of denaturing at 95°C for 15 sec, and annealing and extension at 60°C for 60 sec. The mRNA levels of various genes were calculated after normalizing with β-actin (Figure 1A), or glyceraldehyde 3-phosphate dehydrogenase (GAPDH) (Figure 7), respectively.

### Western blot analysis

Cultured human podocytes were lysed in SDS sample buffer. Protein expression was analyzed by Western blot analysis as described previously [3]. The primary antibodies used were as follows: anti-PINCH1 (#612711; BD Transduction, San Jose, CA), anti-Flag M2 (# F1804; Sigma), anti-GFP (ab290; Abcam, Cambridge, MA), anti-WT1 (sc-192; Santa Cruz Biotechnology, Santa Cruz, CA), anti-podocalyxin (#39-3800; Invitrogen), anti-TBP (TATA binding protein) (ab181–100; Abcam), anti-actin (sc-1616; Santa Cruz Biotechnology) and anti-GAPDH (#4300; Ambion, Austin, TX).

### Immunoprecipitation

Immunoprecipitation experiments were performed using similar methods as described previously [7]. Briefly, human podocyte lysates were centrifuged at 12,000× g for 10 min at 4°C. The supernatants were collected for immunoprecipitation. After preclearing with normal host IgG, the lysates were immunoprecipitated overnight at 4°C with 4 µg antibodies of anti-GFP, anti-Flag, anti-WT1, or the same type of normal rabbit or mouse IgG as controls, followed by precipitation with 60 µl protein A/G Plus-Agarose (Santa Cruz Biotechnology) for 3 h at 4°C. The precipitated complexes were washed three times with lysis buffer and boiled for 5 min in SDS sample buffer, followed by immunoblotting with various antibodies as indicated.

### Nuclear and cytoplasmic fractionation

For preparation of nuclear protein, human podocytes were washed twice with cold phosphate-buffered saline (PBS) and scraped off the plate with a rubber policeman. After centrifugation, cell pellets were resuspended in Buffer A (10 mM HEPES pH 7.9, 1.5 mM MgCl2, 10 nM KCl, 0.5% NP-40 and 1% protease inhibitor cocktail (Sigma)) and lysed with homogenizer. Cell nuclei were collected by centrifugation at 5,000 rpm for 15 min, and the supernatants were saved as cytoplasmic protein preparation. After washing with Buffer B (10 mM HEPES pH 7.9, 1.5 mM MgCl_2_, 10 nM KCl and 1% protease inhibitor cocktail), nuclei were lysed in SDS sample buffer.

### Purification of GST fusion protein and pull down assay

Bacterial BL21 competent cells were transformed with GST and GST-WT1 fusion protein expression vectors, respectively. Bacterial cells were cultured in LB medium containing ampicilin until the OD600 reaches 0.6–0.8, followed by adding 100 µM IPTG to induce recombinant protein expression. After shaking at room temperature for 3 h, cell pellets were collected by spinning at 5,000 *g* for 10 min at 4°C. Bacterial cell lysis was prepared with Rapid GST Inclusion Body Solubilization and Renaturation Kit (Cell Biolabs, Inc., San Diego, CA). Glutathione-agarose beads were used to incubate with bacterial cell lysis overnight at 4°C. Beads were washed three times with PBS containing 1% Triton X-100, and then incubated with podocyte lysates overnight at 4°C. The precipitated complex were washed three times with lysis buffer and boiled for 5 min in SDS sample buffer, followed by immunoblotting with various antibodies as indicated.

### Luciferase reporter assay

The reporter construct pGL3-podocalyxin, which contains the human podocalyxin promoter and the encoding sequence for firefly luciferase, was constructed as described elsewhere [10]. After co-transfection with pGL3-podocalyxin and PINCH1 or/and WT1 expression vectors using Lipofectamine 2000 reagent (Invitrogen), podocytes were incubated for 48 h. The supernatants of cell lysates were collected for the luciferase assay. Luciferase activity was determined using the Dual Luciferase Assay System kit as described by the manufacturer's protocols (Promega). Relative luciferase activity of each group was reported as fold induction over the controls.

### Statistical analysis

All data examined were expressed as mean ± SEM. Statistical analysis was performed using SigmaStat software (Jandel Scientific Software, San Rafael, CA, USA). Comparison between groups was made using one-way analysis of variance (ANOVA), followed by Student-Newman-Keuls test. A *P* value of less than 0.05 was considered significant.
